# Preparation of N/O co-doped porous carbon by a one-step activation method for supercapacitor electrode materials

**DOI:** 10.1039/d2ra02732a

**Published:** 2022-07-21

**Authors:** Dong Liu, Yuling Liu, Yigang Ding, Baomin Fan

**Affiliations:** School of Chemistry and Environmental Engineering, Wuhan Institute of Technology Wuhan 430205 P. R. China; Hubei Key Laboratory of Novel Reactor and Green Chemistry Technology, Wuhan Institute of Technology Wuhan 430205 P. R. China dingyg2016@163.com; Key Laboratory of Processing and Quality Evaluation Technology of Green Plastics of China National Light Industry Council, Beijing Technology and Business University Beijing 100048 China fanbaomin@btbu.edu.cn

## Abstract

Heteroatom-doped carbon materials used in supercapacitors are low in cost and demonstrate extraordinary performance. Here, ethylenediamine tetraacetic acid (EDTA) with intrinsic N and O elements is selected as a raw material for the preparation of heteroatom self-doped porous carbon. Furthermore, N/O self-doped porous carbon with a large surface area has been successfully prepared using K_2_CO_3_ as the activator. The derived sample with a 1 : 2 molar ratio of EDTA to K_2_CO_3_ (EK-2) demonstrates a porous structure, rich defects, a large surface area of 2057 m^2^ g^−1^ and a micropore volume of 0.25 cm^3^ g^−1^. Benefiting from high N content (2.89 at%) and O content (10.75 at%), EK-2 exhibits superior performance, including high capacitance of 325 F g^−1^ at 1 A g^−1^ and outstanding cycling stability with 96.8% retention after 8000 cycles at 10 A g^−1^, which strongly confirms its immense potential toward many applications. Additionally, the maximum energy density of EK-2 reaches was 17.01 W h kg^−1^ at a power density of 350 W kg^−1^ in a two-electrode system. This facile and versatile strategy provides a scalable approach for the batch synthesis of N/O co-doped carbonaceous electrode materials for energy storage.

## Abstract 2

Heteroatom-doped carbon materials used in supercapacitors are low in cost and demonstrate extraordinary performance.

## Graphical abstract

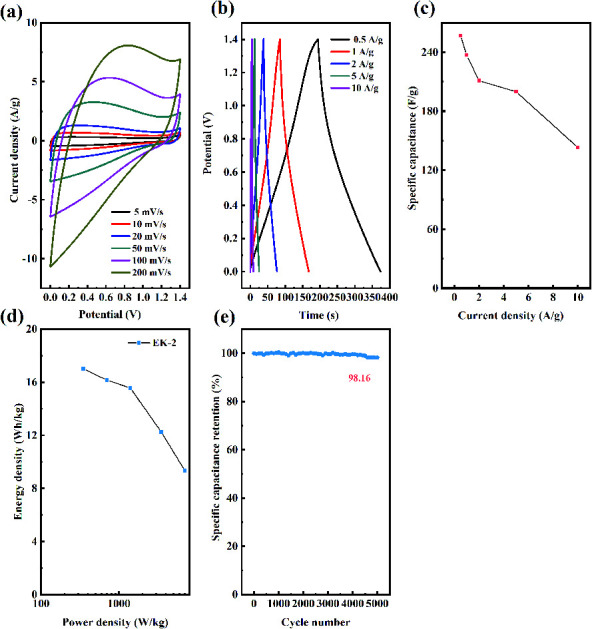

## 1. Introduction

As one of the most attractive energy storage devices, supercapacitors exhibit many advantageous characteristics, such as fast charging–discharging speed, long cycling life, and large specific surface area.^1^ Their performance is largely dependent on the electrode materials. Therefore, many attempts have been made to find cheap and efficient electrode materials suitable for the preparation of high-performance supercapacitors.^2^ Heteroatom doping on the surface or in the bulk of carbon has been a promising strategy to improve capacitance performance. For example, the presence of boron^3^, nitrogen^4^, phosphorus^5^, or sulfur^6^ can confer acid/alkali properties to carbon materials and improve the Faraday interaction between electrolyte ions and functional groups, thereby increasing the specific capacitance through the pseudo capacitance effect.^7^ Among the various heteroatoms, the presence of nitrogen and oxygen atoms can not only enhance the surface tension, wettability, and hydrophilicity of the electrode, but also elevate the inhomogeneity arising from moderate structural defects. In addition, the electron donor characteristics of the carbon matrix can also be optimized. Consequently, close contact between the electrode and the electrolyte is beneficial for facilitating ion acquisition and enhancing the conductivity of the electrode.^8^

Nitrogen doping is one of the most prevalent modification tactics for boosting electrochemical performance. Nitrogen-doping strategies include introducing extra nitrogen from ammonia,^9^ urea,^10^ melamine^11^*etc.*, or using nitrogen-rich substances (poly-aniline^12^, polyacrylonitrile^13^, gelatin^14^*etc.*) as carbon precursors. The former strategy is relatively sophisticated and generally associated with the surface, which leads to poor cycling stability of the electrode. However, the latter directly carbonizes the nitrogen-containing precursors and then further activates them. Nitrogen doping achieved in this way facilitates a more stable structure and superior cycling stability. Nevertheless, the common nitrogen-containing precursors, such as polyaniline and pyrrole, are generally expensive, which limits their large-scale preparation and practical applications. Recently, nitrogen-containing organic salts, such as ethylenediamine tetraacetic acid (EDTA) salts, have been reported as nitrogen-rich carbon precursors (the details are described below). Nitrogen-doped porous carbons with high specific surface area and excellent electrochemical performance can be prepared by one-step pyrolysis without additional cumbersome processes.

In recent years, many researchers have reported a series of nitrogen-containing porous carbons for supercapacitor electrode materials using EDTA as a carbon/nitrogen source. For example, Dai *et al.*^15^ used SBA-15 as a hard template and EDTA-3K as a carbon/nitrogen source to fabricate oxygen and nitrogen-rich materials (8.11 wt% and 2.12 wt%, respectively) with a three-dimensional porous structure. Yang *et al.*^16^ prepared nitrogen-doped porous carbon materials with good performance by the direct pyrolysis of mixtures containing melamine, ferric nitrate, and EDTA, in which EDTA and Fe^3+^ chelates generated macromolecules and retained melamine in the matrix during carbonization. Nitrogen-doped porous carbon was obtained by Xu *et al.*^17^*via* the direct pyrolysis of ethylenediaminetetraacetic acid disodium magnesium salt (EDTANa_2_Mg) under an inert atmosphere, which used a simple process without activation. Owing to the beneficial structural characteristics of the materials, the derived carbon exhibits higher nitrogen content (5.43 at%) and extraordinary capacitance (281 F g^−1^). Using EDTANa_2_Zn as a hard template and nitrogen source, Wang *et al.*^18^ designed and synthesized an optimized electrode material with a porous structure resulting from the dispersion of particles into the nitrogen-doped carbons during pyrolysis, generating plenty of pores. Although the nitrogen content of carbon materials prepared by the above methods is sufficiently high, the specific capacitance tends to be unsatisfactory. Consequently, further development of electrode materials with a high nitrogen content and superb capacitance performance is urgently needed.

Herein, we present a simple method to prepare nitrogen-containing porous carbon materials using EDTA as a carbon/nitrogen source and K_2_CO_3_ as an activator. After mechanically grinding the precursor, the mixture was completely dissolved in a small amount of deionized water. Drying and pyrolysis were then performed to prepare nitrogen-doped porous carbon. Electrochemical tests and material characterizations revealed that the optimal molar ratio of EDTA to K_2_CO_3_ was 1 : 2 (EK-2). Owing to its high specific surface area (2057 m^2^ g^−1^) and rich nitrogen content (2.89 at%), the EK-2 material effectively shortens the diffusion distance of electrolyte ions and allows ions to adequately contact with more active sites. In addition, owing to the intrinsic benefits of the high nitrogen content of EDTA, the physical and chemical properties and capacitance behaviors of the materials were pronouncedly improved.

## 2. Experimental section

### 2.1. Chemicals

The following chemicals were used: EDTA (Analytical Pure, Sinopharm Chemical Reagents Co., Ltd.), K_2_CO_3_ and KOH (Analytical Pure, Fu Chen (Tianjin) Chemical Reagent Co., Ltd.), HCl (Analytical Pure, Xinyang Chemical Reagent Factory), and anhydrous ethanol (Analytical Pure, Wuhan Geo Chemical Technology Co., Ltd.). All experiments were performed using deionized (DI) water. All reagents were of analytical reagent grade and used as received without further treatment.

### 2.2. Preparation of EK-*n* materials

**Fig. 1:**
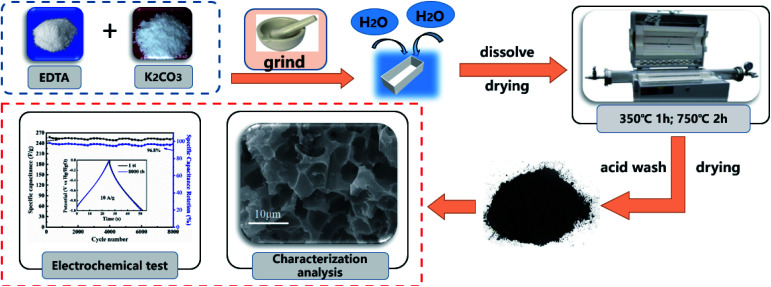
Schematic diagram of the preparation of EK-*n*.

EDTA 2.5 g and different amounts of K_2_CO_3_ (1.18, 2.36, 3.54 and 4.72 g) were ground into a uniform mixture. Then, a small amount of deionized water was added to above mixture and the solution was dried in an oven. After drying, the mixture was transferred to a corundum porcelain boat and then placed in the middle of a quartz tube in a horizontal furnace. The sealed system was heated to 350 °C at a ramp rate of 5 °C min^−1^ under a constant nitrogen flow (20 mL min^−1^). The temperature was held at 350 °C for 1 h, then increased to 750 °C at a heating rate of 5 °C min^−1^ and held for 2 h. The temperature in the horizontal furnace was allowed to cool naturally to room temperature, and the pyrolysis products were collected. Next, to remove the salt residue, the product was soaked in 1 M HNO_3_ solution for 12 h and then washed with deionized water until the pH of the washing solution was neutral. Finally, the samples were obtained for further analysis. The specific preparation process is shown in Fig. 1. The molar ratios of EDTA and K_2_CO_3_ were set as 1 : 1, 1 : 2, 1 : 3, and 1 : 4, and the as-synthetized products are referred to as EK-1, EK-2, EK-3, and EK-4, respectively.

### 2.3. Characterization

Sample morphology was observed by field emission scanning electron microscopy (FE-SEM, Gemini SEM 300, Germany). The microstructural morphology of the samples was studied by transmission electron microscopy (TEM, JEM2010, Japan). Raman spectroscopy was performed with an inVia Raman spectrometer (Renishaw) with 532 nm laser excitation. N_2_ adsorption–desorption isotherms were measured at 77 K (Micromeritics 3Flex, USA). The samples were analyzed using an X-ray diffractometer (XRD, D8 ADVANCE, Germany) and Cu Kα radiation. The surface chemical composition was determined by X-ray photoelectron spectroscopy (XPS, ESCALAB Xi, USA).

### 2.4. Electrochemical measurements

The supercapacitor property of these carbon materials was characterized in a three-electrode system. The working electrode was made of an active material, carbon black and polytetrafluoroethylene (PTFE) in a ratio of 8 : 1 : 1 in ethanol. The as-obtained electrode was pressed onto Ni foam (1.0 cm × 10 cm) and dried at 120 °C for 1 h before pressing at 10 MPa. All electrochemical measurements were performed on a CS350H electrochemical workstation (Wuhan CorrTests Instrument, China) using 6 mol L^−1^ KOH as the electrolyte.

For the three-electrode tests, the Hg/HgO electrode and platinum electrode were used as the reference electrode and counter electrode, respectively. In addition, the electrodes were immersed in 6 mol L^−1^ KOH for 12 h before measurement. The mass loading of the active materials on each working electrode was approximately 2.5–3.0 mg. Cyclic voltammetry (CV) with scan rates from 5 to 100 mV s^−1^ and galvanostatic charge–discharge (GCD) with current densities from 0.5 to 10 A g^−1^ were performed in the potential range of (−1.0 to 0) V in 6 mol L^−1^ KOH electrolyte. Electrochemical impedance spectroscopy (EIS) was between 100 kHz and 0.01 Hz with an amplitude of 10 mV. In the three-electrode system, the specific capacitances of electrodes (*C*, F g^−1^) were calculated by eqn (1):1
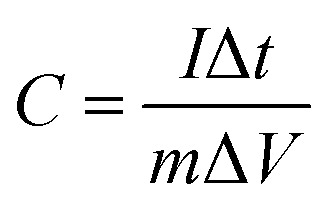
where *C* denotes specific capacitance (F g^−1^); *I* is the discharge current (A); Δ*t* is the discharge time (s); *m* is the mass of the active substance on the electrode (g), and Δ*V* is the working voltage window (V).

As for the two-electrode system, the mass loading of the active materials on each working electrode was approximately 2.88 mg. The two-electrode system consisted of working electrodes with the same load, separated by filter paper. The whole system was completely immersed in electrolyte (6 mol L^−1^ KOH). The system was evaluated over a voltage window of (0–1.4) V in 6 M KOH electrolyte. The specific capacitance of the two-electrode system was determined from the following equations:^19^2
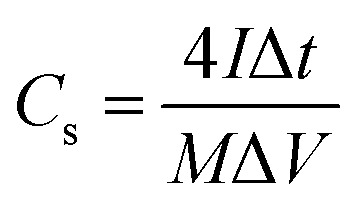
3
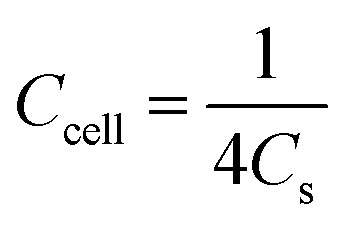
4
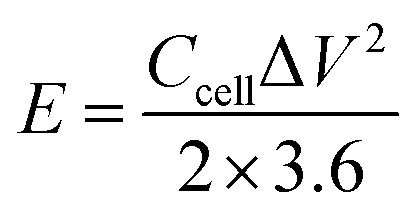
5
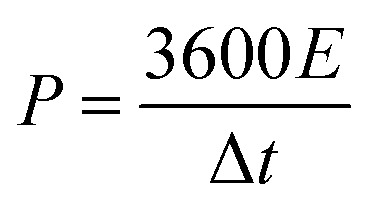


## 3. Results and discussion

The synthesis of porous carbon from ethylenediamine tetraacetic acid and potassium bicarbonate is illustrated in Fig. 1. First, ethylenediamine tetraacetic acid and potassium carbonate were acid–base neutralized to form potassium-containing compounds during the grinding process at room temperature. Subsequently, by changing the molar ratio of ethylenediamine tetraacetic acid and potassium carbonate, we obtained a range of compounds with different potassium content. Subsequently, calcination was performed through a one-step activation method and the resulting products were treated with dilute acid to generate porous carbon materials.

**Fig. 2:**
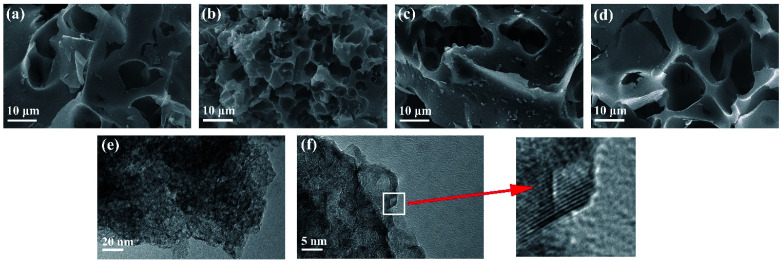
Characterization of EK-*n* samples: SEM images of (a) EK-1, (b) EK-2, (c) EK-3, and (d) EK-4, and (e and f) TEM images of EK-2 with the inset of the HRTEM image.

The morphology and microstructure of the prepared carbon were characterized by field emission-scanning electron microscopy (SEM) and transmission electron microscopy (HRTEM). The morphology of samples activated by different amounts of K_2_CO_3_ is presented in Fig. 2. As demonstrated in Fig. 2a, the sample presented porous structure and thin carbon layer, which are attributed to the sufficient interaction between the precursor and K_2_CO_3_ during the annealing process. As the K_2_CO_3_ increases, smaller pores and curved structures can be observed in Fig. 2b, indicating the formation of thinner lamella and numerous porous structures. As the pores increased, the morphology of EK-2 gradually evolved into a porous honeycomb structure, and the lamellar thickness decreased significantly. With further addition of K_2_CO_3_ addition (molar ratio > 2), some of the micropores in EK-3 and EK-4 were transformed into mesopores. In addition, the collapse of the porous structure results in stackable fragment structures, as shown in Fig. 2c and d. Extensive areas of mesoporous were produced owing to the accumulation of fragments. The change in morphology may be caused by the excessive redox reaction between EDTA and K_2_CO_3_, the potassium metal can be easily inserted into the carbon lattice structure and partially exfoliates the carbon layer.^20^

We also performed HRTEM analysis of EK-2. From Fig. 2e and f, it can be seen that the surface of EK-2 is covered with a wormlike microporous structure, and some serrated edges are distributed throughout the ultra-thin layer structure. In addition, some small graphite stripes can be observed (Fig. 2f), indicating that the degree of graphitization is improved, which is beneficial to the conductivity of carbon materials.^21^ The formation of the above pore size is due to the addition of the K_2_CO_3_ activator, during the high temperature (∼700 °C) treatment of K_2_CO_3_, a certain amount of compounds can be generated, such as K_2_O, CO and CO_2_. Firstly, the K vapors can intercalate into carbon materials, making swelling and disruption of carbon microstructure and creating additional porosity. And then the formation of CO and CO_2_ can facilitate the development of porosity behavior through carbon gasification. The reaction can be listed as follows (eqn (6)–(9)):^22^

6 K_2_CO_3_ → K_2_O + CO_2_

7 K_2_CO_3_ + 2C → 2K + 3CO

8 CO_2_ + C → 2CO

9 C + K_2_O → 2K + CO

**Fig. 3:**
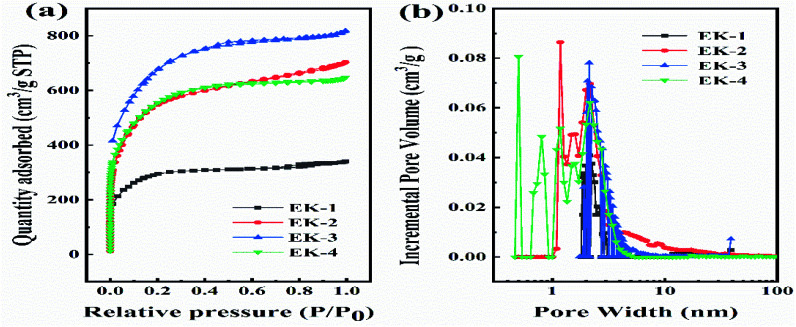
Characterization of EK-*n* samples: (a) N_2_ adsorption isotherm, and (b) pore size distribution.

The specific surface area and pore size distribution of the samples were determined *via* the nitrogen adsorption/desorption isotherm measurement. As shown in Fig. 3a, the adsorption capacity of EK-*n* enhanced rapidly with an increase in relative pressure under low relative pressure (*P*/*P*_0_). When the relative pressure approached zero, the isotherm had a sharp upward trend due to the presence of micropores (type I features). Furthermore, from 0.4 to 1.0*P*/*P*_0_, there is a significant hysteresis loop, indicating the presence of mesoporous or macroporous structures in the sample (representing type IV). As depicted in Fig. 3a, with the increase in relative pressure, the adsorption capacity sharply increases under relatively low pressure (*P*/*P*_0_ < 0.1), which is indicative of an abundance of micropores in the sample. A small hysteresis ring appeared in the medium pressure range of 0.5–0.9, revealing that micropores are formed as a result of the increase in K_2_CO_3_, which in turn accumulated and develops into mesopores or macropore.^23^ As displayed in Fig. 2b, there was a peak in the micropore range, locating at 1.18 nm. Similarly, there was a peak in the mesoporous range, situating at 2.17 nm. Apparently, the two peaks provided significant evidence for the rich micro/mesoporous structure of EK-2.

The porosity parameters for EK-*n* are given in Table 1. The specific surface area of the sample increased with the increasing of the amount of activator potassium carbonate. However, when the potassium carbonate was further increased to a certain amount (molar ratio of 1 : 4), the structure collapsed due to excessive etching effect of activator, which might lead to the decrease of the specific surface area. Although EK-3 has the largest specific surface area (2379 m^2^ g^−1^), it does not provide sufficient active sites because of the small microporous surface area (379 m^2^ g^−1^), which limits the accessibility of electrolyte ions. Even though the specific surface area of EK-2 is not the largest (2057 m^2^ g^−1^), it still has the largest micropore area (716 m^2^ g^−1^), total pore volume (1.09 cm^3^ g^−1^), and micropore volume (0.25 cm^3^ g^−1^), which confirmed the results of the SEM characterization. Related studies have confirmed that a rich microporous structure is crucial for energy storage owing to its enhancement of the specific surface area.^18,24^ Moreover, it can also boost the specific capacitance by introducing a substantial number of active sites. Simultaneously, the mesoporous structure can effectively shorten the ion diffusion path, facilitating electrolyte penetration, and support the high utilization rate of the microporous surface. In short, high specific surface area and porous (micro/mesoporous) structure are pivotal for capacitance performance.^25^

**Table 1:** Porosity parameters of EK-*n*

Sample	*S*_BET_ a (m^2^ g^−1^)	*S*_mic_ a (m^2^ g^−1^)	*V*_total_ a (cm^3^ g^−1^)	*V*_mic_ a (cm^3^ g^−1^)	N^b^ (at%)	O^b^ (at%)	Capacitance^c^ (F g^−1^)
EK-1	1005	409	0.53	0.19	1.95	13.52	281
EK-2	2057	716	1.09	0.25	2.89	10.75	325
EK-3	2379	379	1.27	0.17	1.97	7.86	265
EK-4	2037	379	1.00	0.14	2.67	7.01	284

a Calculated using a multi-point BET method.

b N, O represent their atomic content.

c The above specific capacitance represents the specific capacitance at a current density of 1 A g^−1^.

**Fig. 4:**
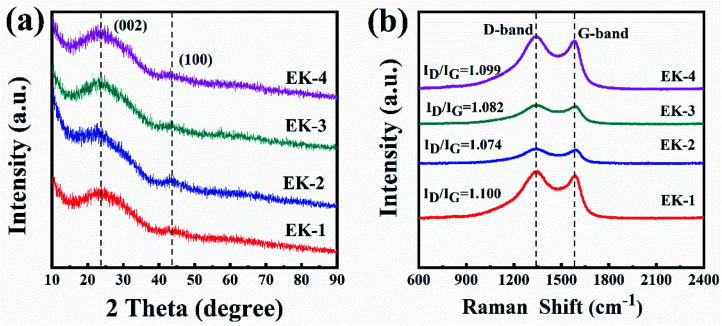
Characterization of EK-*n* samples: (a) XRD spectra, and (b) Raman spectra.

XRD patterns and Raman spectra were used to characterize the graphitic properties of the EK-*n* samples in Fig. 4. In the XRD patterns (Fig. 4a), two diffraction peaks located at ∼24° and ∼44° are shown, corresponding to the diffraction of the (002) and (100) planes of graphitic carbon, respectively. Moreover, all samples could be observed, indicating the successful preparation of carbons and the presence of a graphitic structure in the samples.^21,26^ Compared with other materials, the shape of the (002) peak of EK-2 is relatively strong and slightly narrow, indicating an increased degree of graphitization.^22^ The Raman spectra are presented in Fig. 4b. The two major peaks at approximately 1343 cm^−1^ and 1587 cm^−1^ were assigned to the D and G bands respectively. The D band corresponds to the imperfections in graphite or the lattice disorder in the sp^3^ carbon. The G band is associated with the sp^2^ carbon and corresponds to the crystalline graphite.^27^ The ratio of the intensity of the D band to the G band (*I*_D_/*I*_G_) can indicate the graphitization degree of carbon.^28^ The intensity ratio of the EK-2 sample is the smallest (1.074), confirming that the sample EK-2 has the less amorphous structure and higher graphitization degree. Relevant studies have shown that a certain degree of graphitization can enhance the electrical conductivity of ions, thereby increasing the specific capacitance,^29^ consistent with the XRD results.

**Fig. 5:**
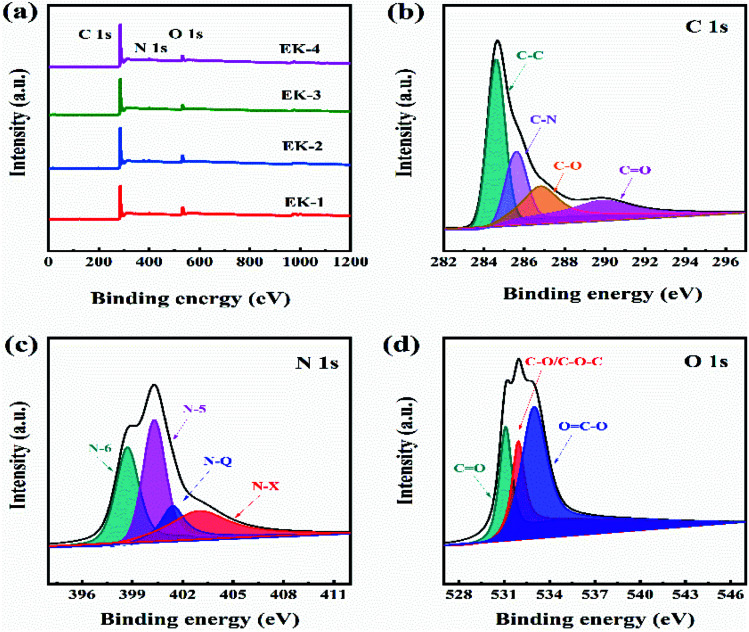
(a) XPS survey of EK-*n* samples; (b) C 1s, (c) N 1s, and (d) O 1s high-resolution spectra of EK-2 sample.

XPS was used to investigate the atom content and elemental chemical states in the EK-2 sample. XPS analysis confirmed the presence of carbon, nitrogen, and oxygen atoms in the EK-2 sample as 86.36 at%, 2.89 at%, and 10.75 at%, respectively (as shown in Fig. 5a). In the XPS spectrum of C 1s of EK-2, the four sub-peaks could be attributed to C–C, C–N, C–O, and C=O at 284.6, 285.6, 286.8, and 289.9 eV,^30^ respectively (Fig. 5b). Abundant nitrogen-containing functional groups were found in sample EK-2, which exhibits four different types of nitrogen atoms: pyridinic nitrogen (N-6, 398.7 eV), pyrrolic nitrogen (N-5, 400.3 eV), quaternary nitrogen (N–Q, 401.4 eV), and pyridinic-nitrogen-oxides (N–X, 403.0 eV) (as shown in Fig. 5c). The positively charged N–Q and N–X in the carbon matrix could improve the electron transfer ability and effectively enhance the conductivity of the electrode material, whereas the negatively charged N-6 and N-5 could provide additional free or delocalized electrons to the electron-deficient carbon atoms, thereby producing additional pseudocapacitance.^8,31^ The O 1s spectra of the materials (Fig. 5d) were used to investigate the type of oxygen-containing groups. Three types of oxygenated groups were found: C=O (531.2 eV), C–OH/C–O–C (532.15 eV), and O=C–O (532.97 eV).^32^ The relative contribution of C=O is the highest. C=O and other oxygen-containing functional groups play active roles in the Faraday reactions during charge/discharge processes.^33^ The presence of N and O groups can improve the surface tension, wettability, and hydrophilicity of carbon materials. These properties are conducive to the close contact between the electrode and the electrolyte. Furthermore, the acquisition of electrolyte ions can also be enhanced, leading to an increase in electrical conductivity.^8^

We prepared EK-*n* electrodes and subjected them to electrochemical testing at room temperature using 6 mol L^−1^ KOH solution as the electrolyte to study their electrochemical performance as an electrode material for supercapacitors.

**Fig. 6:**
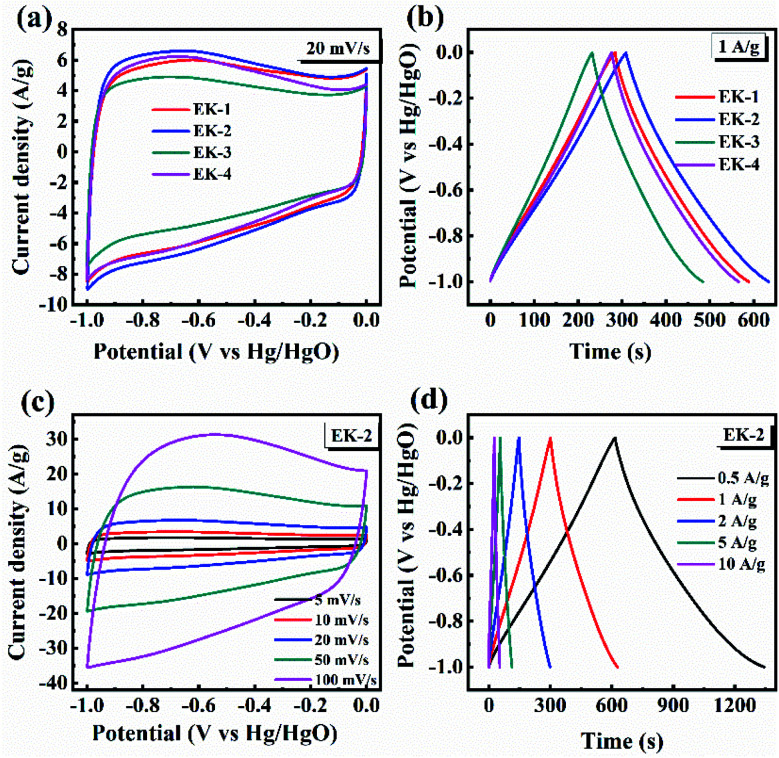
Supercapacitive performance tested by three-electrode configuration in 6.0 mol L^−1^ KOH: (a) CV plot at 20 mV s^−1^ of EK-*n*; (b) GCD curves at 1 A g^−1^ of EK-*n*; (c) CV plots of EK-2 at various scan rates; and (d) GCD plots of EK-2 at various current densities.

The CV curves of the prepared EK-*n* electrodes measured at a scan rate of 20 mV s^−1^ are presented in Fig. 6a. As shown in CV plots, the curves of all samples were rectangular-like in shape and accompanied by redox peaks, indicating that the capacitance of samples consisted of electric double-layer capacitance (EDLC) and pseudocapacitance (PC), in which the pseudocapacitance was derived from the reversible reaction of electrochemically active nitrogen and oxygen with the electrolyte.

In the above XPS tests, the nitrogen functional groups are mainly found in the form of pyridinic nitrogen (N-6), pyrrolic nitrogen (N-5), quaternary nitrogen (N–Q), and pyridinic-N-oxide (N–X), which are all electroactive functional groups able to provide additional pseudocapacitance.^34^ For the oxygenated functional groups (C=O and C–OH), a reversible redox reaction can occur, this can be represented as –C=O + H^+^ + e^−^ ↔ –C–OH. This reaction can also generate pseudocapacitance. Relevant studies have revealed that oxygen-containing functional groups (C=O, C–OH, and –OH) on the surface of materials can increase the effective contact area between electrode surface and carbon materials,^31^ and enhance surface polarity, which are beneficial to pseudocapacitance formation.^35^ It is clear that EK-2 has the largest enclosed area, with a faster ion diffusion rate and larger ion-accessible specific surface area.^36^ The galvanostatic charge/discharge curves (GCD) of the prepared EK-*n* electrode at a current density of 1 A g^−1^ are shown in Fig. 6b. The GCD curves of all samples are approximately isosteric triangles and indicate superior electrochemical reversibility. Moreover, the EK-2 electrode has the longest charge–discharge time, indicating it has best capacitive performance. As illustrated in Fig. 6c, all the CV curves still maintain a quasi-rectangular shape when the scan rate is increased to 100 mV s^−1^, signifying that low polarization with fast response of ion transportation occurred in the EK-2 electrode. The GCD curve of EK-2 under different current densities (0.5–10 A g^−1^) is shown in Fig. 6d. The charge–discharge time of EK-2 gradually shortens with the increase in current density, corresponding to the decrease in specific capacitance. The reason may be that the charge diffusion fails to match the rapidly increasing current density consequently, the electrode material cannot be filled by electrolyte ions.^37^

**Fig. 7:**
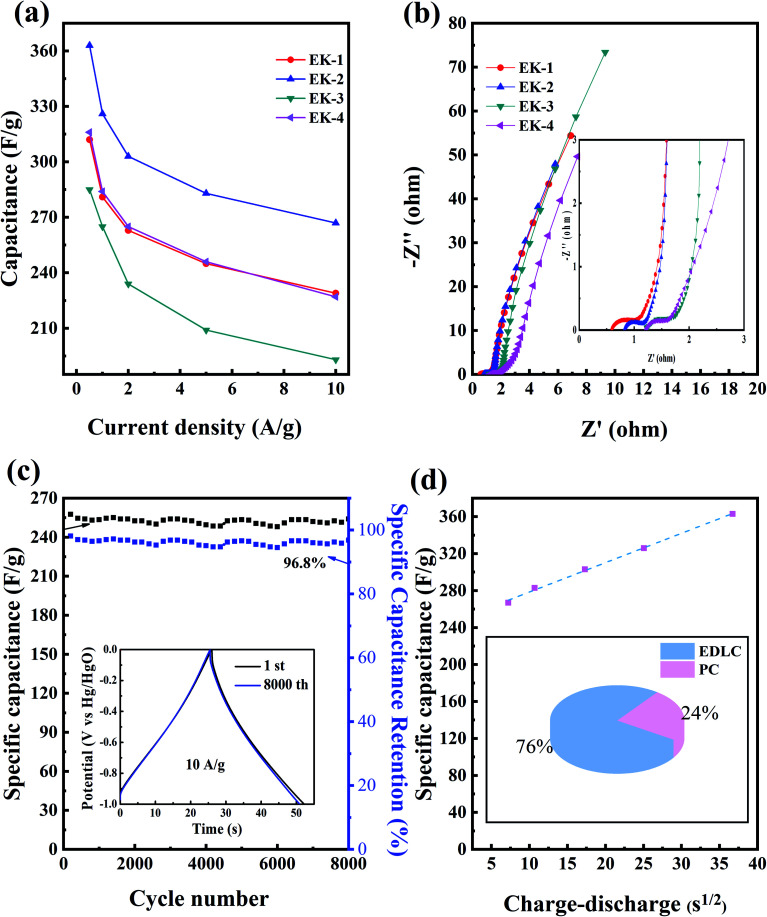
Supercapacitive performance tested by the three-electrode configuration in 6.0 M KOH: (a) capacitance at different current densities of EK-*n*; (b) Nyquist plots of EK-*n*; and (c) cycle durability at a current density of 10 A g^−1^ for 8000 cycles with charge–discharge curves, with the inset showing the first and last constant current charge–discharge curves for EK-2. (d) Trasatti analysis results: capacitance *versus* square root of the charge–discharge time for the three-carbon electrode for EK-2.

The EK-*n* electrode materials at different current densities (0.5–10 A g^−1^) of the discharge-specific capacitances are shown in Fig. 7a. When the current density was 1 A g^−1^, the specific capacitances of the EK-1, EK-2, EK-3, and EK-4 samples were 281 F g^−1^, 325 F g^−1^, 265 F g^−1^, and 284 F g^−1^. When the current density was increased to 10 A g^−1^, the specific capacitances of the five samples were 229 F g^−1^, 267 F g^−1^, 193 F g^−1^, and 227 F g^−1^, respectively. Notably, the EK-2 sample exhibits characteristics of galvanostatic charge/discharge at large current densities and exceptional rate capability (82% retention at 1–10 A g^−1^) compared with other porous carbon materials prepared from EDTA derivatives published in the literature (shown in Table 2).

**Table 2:** Comparison of electrochemical performance of porous carbons tested in a three-electrode cell

Materials	Specific capacitance	Measurement condition	Electrolyte	Ref.
3DHPNCF-700	213 F g^−1^	1.00 A g^−1^	6 M KOH	15
NC-700	275 F g^−1^	0.30 A g^−1^	6 M KOH	16
ENM700	281 F g^−1^	0.05 A g^−1^	6 M KOH	17
ESC950	311 F g^−1^	0.10 A g^−1^	1 M H_2_SO_4_	18
LSAC-3	499 F g^−1^	0.10 A g^−1^	6 M KOH	41
EK-2	325 F g^−1^	1.00 A g^−1^	6 M KOH	This work

We also performed EIS tests, as shown in Fig. 7b. An electrochemical resistive map can effectively analyze the internal resistance and interfacial resistance of electrode materials related to electrolytes, obtaining to further evaluation of their electrochemical behavior.^38^ Four samples showed impedance spectra similar to a semicircle in the high-frequency region, which corresponds to an electron-limited process with a radius equivalent to faradaic charge transfer resistance (*R*_ct_). The straight line at the low frequency indicated the ideal behavior of the EDLC, which was more consistent with the CV curve.^39^ The solution resistance (*R*_s_) is the intersection point between the high-frequency range and the real *Z*_0_: for each sample, the *R*_s_ value was small. Collectively, EK-2 has the smallest semicircle impedance loop in the high-frequency region, which indicates a low charge transfer resistance (*R*_ct_) and an almost straight line in the low-frequency region (close to the longitudinal axis); this shows that, electrolyte ions diffuse rapidly and are rapidly adsorbed on the electrode surface. Consequently, these phenomena indicate that the diffusion and transfer of ions from the electrolyte to the pores occur more easily, which creates favorable conditions for the increase in specific capacitance.^40^

The EIS test results are consistent with the cyclic voltammetry and galvanostatic charge–discharge test results. As seen from Fig. 7c, the electrochemical stability plot shows that the capacitance retention of EK-2 at a current density of 10 A g^−1^ after 8000 charge–discharge cycles still has 96.8% compared with the initial specific capacitance. Meanwhile, it can be seen from the inset that the galvanostatic charge and discharge patterns of the first cycle and the last cycle can be considered nearly coincident in the cycling stability test, providing further indication of the high cycling stability of the EK-2 sample. This is consistent with the conclusions drawn from the cyclic volt–ampere curve and the constant current charge–discharge curve. This result confirms that the EK-2 sample has favourable and competitive long-term cycling stability and can be used as an electrode material for supercapacitors. To estimate the specific contributions of EDLC and pseudocapacity, capacitance was plotted against the square root of the half-cycle time (Fig. 7d). The intersection of the dotted line with the vertical axis denotes the rate-independent capacitance,^34,42,43^ indicating that the EDLC contribution is 247 F g^−1^ for EK-2. The pseudocapacitance contribution of the N/O species is approximately 78 F g^−1^ for EK-2 (covering 24% of total capacitance). As far as EDTA is concerned, it contains two kinds of heteroatoms, nitrogen and oxygen atom, which can be confirmed from XPS characterization. There's research that proves these N and O may be converted into O- or N-containing functional groups in the carbon matrix after thermal treatment. These functional groups are reduced upon interaction with H^+^ or OH^−^ ions in the aqueous electrolyte. This charge transfer reaction provides additional faradaic reactions by which to store electrical charge. Possible reactions are as follows eqn (10)–(14):^8,33^

10 =CH–NH_2_ + 2OH^−^ ↔ =C=NH + 2H_2_O + 2e^−^

11 =CH–NH_2_ + 2OH^−^ ↔ C–OHNH + H_2_O + 2e^−^

12 =C–OH + OH^−^ ↔ =CO^−^ + H_2_O

13 =C=O + OH– ↔ –COOH + e^−^

14 –COOH + OH^−^ ↔ –COO^−^ + H_2_O

**Fig. 8:**
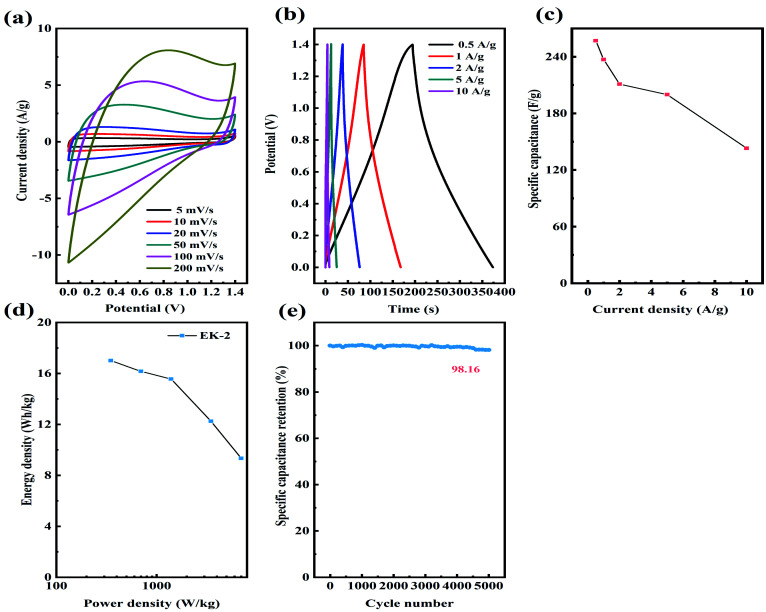
The supercapacitive behaviors of the symmetric EK-2 supercapacitor: (a) CV curves at different scan rates; (b) various current densities of GCD curves; (c) specific capacitance; (d) Ragone plot; (e) cyclic stability of the device.

Considering the superior supercapacitive performance exhibited by EK-2 in the three-electrode evaluation, we can reasonably infer that when assembled on a symmetric supercapacitor (EK-2//EK-2), it will generate a robust power output. The CV and GCD curves of supercapacitors in the potential range of 0–1.4 V were measured in 6 mol L^−1^ KOH. A quasi-rectangular form was obtained in the EK-2-based supercapacitor CV curves (Fig. 8a) and a Faraday hump was clearly observed over the range of 0.4–1.2 V to provide partial pseudocapacitance due to the nitrogen doping.^44^ Notably, even at a scan rate of 200 mV s^−1^, the CV curves retained almost their initial form, suggesting the excellent capacitive conductivity and rate performance of the EDLC.^45,46^ Furthermore, a symmetric triangular shape was observed in the GCD curves of the EK-2-based supercapacitor at various GCDs from 0.5–10 A g^−1^ (Fig. 8b), illustrating remarkable electrochemical reversibility. The EK-2 based symmetric super-capacitor could deliver a high *C*_s_ of 257 F g^−1^ with a retention ratio of 55.6% at 10 A g^−1^ in 6 M KOH solution (Fig. 8c), demonstrating the good rate capability. The Ragone plots of EK-2-based symmetric supercapacitors are displayed in Fig. 8d. The EK-2//EK-2 has a maximum energy density of up to 17.01 W h kg^−1^ with a power density of 350 W kg^−1^. As shown in Table 3, this value is extremely competitive compared with some reported heteroatom-doped symmetric supercapacitors. As illustrated in Fig. 8e, the rate capability of the symmetric device was remained at 98.16%, the EK-2 electrode proved its high stability through 5000 cycles with a small degradation.

**Table 3:** Comparison of energy density and power density performance of porous carbon in two-electrode batteries

Sample	Energy (W h kg^−1^)	Power density (W kg^−1^)	Ref.
BNAC-3	12.00	375.0	44
NC-HAP-700	11.90	600.0	47
LDC	12.20	86.8	48
PGBC	6.68	100.2	49
NSG-210	8.30	510.0	50
EK-2	17.01	350.0	This work

The excellent electrochemical efficiency of EK-2 can be attributed to the following points: (1) a large collection of active adsorption sites can be increased by the high specific surface and micropore surface region, ensuring the contribution of EDLC to the total capacitance. The broad range of pore volumes and well-developed layered pore structure can supply ample pore channels and can facilitate the ion transport *via* shortened pathways. (2) The coexistence of O and N functional groups in the carbon matrix improves the wettability and electrical conductivity of carbon. In addition, reversible redox reactions are involved, which generate a large pseudocapacitance that significantly enhance electrochemical activity.

## 4. Conclusions

To summarize, N/O co-doped porous carbon materials were successfully prepared by the one-step pyrolysis of EDTA and K_2_CO_3_. A range of test results indicated that the EK-2 displays a large specific surface area (2057 m^2^ g^−1^) and microporous structure (716 m^2^ g^−1^) that provides significant amounts of active sites for electrolyte ion diffusion. Besides, the existence of nitrogen and oxygen (2.89 at% N and 10.75 at% O) in EDTA can greatly improve the electrochemical performance. As expected, EK-2 exhibits favourable specific capacitance of 325 F g^−1^ at 1 A g^−1^, outstanding cycle performance (only a 3.2% loss over 8000 cycles), and robust rate capability (82% capacitance retention at 1–10 A g^−1^). In particular, the EK-2-based symmetric supercapacitor with a wide voltage window 0–1.4 V can offer a high energy density of 17.01 W h kg^−1^ at a power density of 350 W kg^−1^. The fabrication strategy might be applicable for the grafting of electrochemically active nitrogen species on other types of carbon nanostructures and thus expanding the potential for application in various fields, such as sorption, catalysis, and sensing.

## Conflicts of interest

There are no conflicts to declare.

## Supplementary Material
